# Hierarchical Control of Nitrite Respiration by Transcription Factors Encoded within Mobile Gene Clusters of *Thermus thermophilus*

**DOI:** 10.3390/genes8120361

**Published:** 2017-12-01

**Authors:** Laura Alvarez, Nieves G. Quintáns, Alba Blesa, Ignacio Baquedano, Mario Mencía, Carlos Bricio, José Berenguer

**Affiliations:** 1 Centro de Biología Molecular Severo Ochoa, Universidad Autónoma de Madrid—Consejo Superior de Investigaciones Científicas, 28049 Madrid, Spain; laura.alvarez@umu.se (L.A.); ngquintans@cbm.csic.es (N.G.Q.); ablesa@cbm.csic.es (A.B.); ibaquedano@cbm.csic.es (I.B.); mmencia@cbm.csic.es (M.M.); carlos.bricio@gmail.com (C.B.); 2 Current Address: Laboratory for Molecular Infection Medicine Sweden, Department of Molecular Biology, Umeå University, 90187 Umeå, Sweden

**Keywords:** denitrification, nitrite respiration, regulation, thermophiles, *Thermus thermophilus*

## Abstract

Denitrification in *Thermus thermophilus* is encoded by the nitrate respiration conjugative element (NCE) and nitrite and nitric oxide respiration (*nic*) gene clusters. A tight coordination of each cluster’s expression is required to maximize anaerobic growth, and to avoid toxicity by intermediates, especially nitric oxides (NO). Here, we study the control of the nitrite reductases (Nir) and NO reductases (Nor) upon horizontal acquisition of the NCE and *nic* clusters by a formerly aerobic host. Expression of the *nic* promoters *PnirS*, *PnirJ*, and *PnorC*, depends on the oxygen sensor DnrS and on the DnrT protein, both NCE-encoded. NsrR, a *nic*-encoded transcription factor with an iron–sulfur cluster, is also involved in Nir and Nor control. Deletion of *nsrR* decreased *PnorC* and *PnirJ* transcription, and activated *PnirS* under denitrification conditions, exhibiting a dual regulatory role never described before for members of the NsrR family. On the basis of these results, a regulatory hierarchy is proposed, in which under anoxia, there is a pre-activation of the *nic* promoters by DnrS and DnrT, and then NsrR leads to Nor induction and Nir repression, likely as a second stage of regulation that would require NO detection, thus avoiding accumulation of toxic levels of NO. The whole system appears to work in remarkable coordination to function only when the relevant nitrogen species are present inside the cell.

## 1. Introduction

Many bacteria, archaea, and a few fungi species are able to utilize nitrogen oxides (NO_x_) as electron acceptors at low oxygen concentrations [1]. In this process, known as denitrification, water-soluble nitrate and nitrite are eliminated from the local environment through conversion into gaseous nitrous oxide (N_2_O) or dinitrogen (N_2_) that escape to the atmosphere [2,3,4]. The conversion of nitrate to dinitrogen is carried out in four steps (nitrate > nitrite > nitric oxide > nitrous oxide > dinitrogen) by the corresponding membrane-bound or periplasmic reductases (Nar/Nap, Nir, Nor, and Nos). All four reductases are found in complete denitrifying microorganisms, such as *Pseudomonas* spp. and *Paracoccus denitrificans*, employed as models of the whole denitrification process [1,5,6,7,8,9].

In well-characterized denitrifying bacteria, the genes encoding the denitrification reductases are transcribed in response to two signals: low oxygen levels, and presence of the appropriate nitrogen oxide species [7]. Low oxygen is most frequently signaled in denitrification by homologues of fumarate and nitrate reduction regulatory protein (FNR) a transcription factor of the catabolite repressor protein (CRP) family that contains an oxygen-sensitive [4Fe-4S] iron–sulfur cluster, or by heme-containing proteins of the FixL family, whereas nitrate and nitrite are detected through two-component systems of the NarX/NarL type. Downstream enzymes of the denitrification pathway are expressed upon detection of NO, driven by a specialized and diverse set of sensory transcription factors [10]. Proteins of the CRP family, designated as NNR and DNR, sense NO through an *N*-terminal domain containing a heme group, and are the main NO sensors in *Pseudomonas* spp. and related bacteria [11,12]. Proteins of the NorR family, present in diverse bacteria, sense NO likely through nitrosylation of a mono-nuclear non-heme iron present in a *N*-terminal cGMP-specific phosphodiesterases, adenylyl cyclases and FhlA (GAF) domain. Finally, members of the NsrR family regulate the expression of NO detoxifying enzymes or the expression of denitrification genes in different Gram-positive and Gram-negative bacteria, by an *N*-terminal [4Fe–4S] cluster that is oxidized to a 2Fe–2S cluster upon exposure to O_2_, and likely to NO, abolishing its DNA binding capability [13].

The genus *Thermus* includes hundreds of aerobic or facultatively anaerobic isolates, of which the *Thermus thermophilus* HB8 and HB27 strains have been extensively used as laboratory models, due to their fast growth and highly efficient natural competence system [14], facilitating the development of a complete genetic toolbox [15]. These two strains are obligate aerobes, and their respiratory complexes are well known, even at the structural level [16,17,18,19,20,21]. However, many environmental *Thermus* isolates grow anaerobically by reducing nitrate to nitrite, or by carrying out almost complete denitrification, N_2_O being the final product [22,23,24]. Transformation efficiencies of these environmental denitrifying strains is quite low, and therefore, in order to ease the study of the denitrification process, the gene clusters encoding denitrification enzymes were transferred to the *T. thermophilus* HB27 strain, both by transformation and by “transjugation”, a cell-to-cell transfer mechanism unrelated to common conjugation [23,25,26]. In contrast with the natural denitrifying strains, the derivative *T. thermophilus* HB27d shows high transformation efficiency, allowing for the generation of directed insertion mutants needed for the analysis of the denitrification pathway in extreme thermophiles.

As observed in the natural donor strains, the genes encoding denitrification in *T. thermophilus* HB27d localize to a highly variable region of the pTT27 megaplasmid. Nitrate respiration is encoded within a 30-kbp genetic island named the nitrate respiration conjugative element (NCE) [23,27], and includes operons for a nitrate reductase and nitrate/nitrite transporters (*narCGHJIKT*), signaling, and regulatory proteins (*dnrST*, *drpAB*), and a dedicated type II NADH dehydrogenase (*nrcDEFN*). Nitrite respiration is encoded by the nitrite and nitric oxide respiration (*nic*) cluster, a 7-kb region separated from the NCE and surrounded by insertion sequences [24]. The *nic* cluster encodes NirS, a cd_1_-type nitrite reductase, a protein likely involved in its maturation (NirJ), an electron transporter (NirM) [28], a heterotrimeric NO reductase (NorCBH) [29] and putative regulatory genes (*nsrRST*).

Nitrate respiration in *T. thermophilus* HB27d also responds to low oxygen and nitrate, but scarcely to nitrite [24]. Although no homologues of FNR or NarX/NarL exist in its genome [24], the sensory-signaling system for nitrate and anoxia in *Thermus* spp. is genetically linked to the NCE [27,30]. Two proteins (DnrS and DnrT) encoded in a bicistronic operon upstream of the nitrate reductase were identified as essential for the transcription of the nitrate reductase. DnrS is a large cytoplasmic protein with an *N*-terminal GAF and a *C*-terminal bacterial transcriptional activator domain (BTAD) domains, which is required for transcriptional activation of the *nar* and *dnr* operons, but not for expression of *nrc* [30]. As oxygen induces conformational changes in DnrS that make it highly susceptible to proteases [30], we hypothesized that it functions as the systemic oxygen sensor, through a yet unknown mechanism related to its GAF domain. However, every attempt to obtain biochemical data on its function, in vitro, has failed, due to difficulties expressing an active form in *Escherichia coli*. On the other hand, DnrT belongs to the DNR subgroup of the CRP family of transcription activators [31], and contains a C-terminal helix-turn-helix (HTH) DNA binding motif, and a putative *N*-terminal cyclic nucleotide binding motif, but lacks the cysteine motif required for incorporation of an iron-sulfur cluster as in FNR. DnrT is insensitive to oxygen, and functions as a transcriptional activator required for the expression of its own operon, and for that of the *nar* and *nrc* operons [30]. Though electrophoretic mobility shift assays (EMSA) with DnrT have been unsuccessful, in vitro transcription assays with the *nrc* operon promoter have revealed that DnrT recruits the *T. thermophilus* RNA polymerase, allowing the identification of its binding site [30].

DnrS and DnrT are not just local regulators acting at the level of the NCE; DnrT also functions as a transcriptional repressor of the chromosomal *nqo* and *fbc* operons, encoding the respiratory complexes I and III, respectively [22,30], consequently controlling the transition from aerobic respiration to denitrification [30]. Moreover, derivative mutants of complete denitrifying strains lacking either DnrS or DnrT are unable to grow with nitrite as electron acceptor, supporting that they are also required for the expression of the nitrite and/or NO reductases [22].

In this work, we study the regulation of the promoters in the *nic* nitrite respiration cluster. Our results demonstrate that DnrS and DnrT, the denitrification master regulators, and a *nic*-encoded local regulator of the NsrR family (named NsrR in *Thermus* as well), act in concert to control the expression of the Nir and Nor reductases in *T. thermophilus*. These data support that genetic regulation of denitrification in the ancient genus *Thermus* spp. significantly differs from that found in modern mesophilic bacteria.

## 2. Materials and Methods

### 2.1. Bacterial Strains and Growth Conditions

Bacterial strains used in this work are described in Table 1. Aerobic growth of *T. thermophilus* strains was carried out at 70 °C with rotational shaking (Thermos Scientific, MAXQ 420 HP, 150 rpm) in flasks filled up to 1/5 of capacity with *Thermus* broth (TB) [32]. Anaerobic growth was achieved in screw-capped tubes containing 10 mL of TB supplemented with potassium nitrate (20 mM), sodium nitrite (5 mM), or sodium nitropruside (SNP) (100 μM), and overlaid by mineral oil. *T. thermophilus* colonies were grown aerobically on *Thermus* agar (1.5% *w/v* agar) plates. For liquid or solid selection, kanamycin (30 mg L^−1^), hygromycin B (100 mg L^−1^), and/or bleomycin (15 mg L^−1^) were added when required.

*E. coli* strain DH5*α* was used for construction of plasmids, whereas *E. coli* strain BL21(DE3) was used for overexpression and purification of recombinant proteins. *E. coli* was grown at 37 °C in liquid or solid lysogeny broth (LB) media, with kanamycin (30 mg L^−1^), ampicillin (100 mg L^−1^), hygromycin B (100 mg L^−1^), or bleomycin (3 mg L^−1^) added when required.

### 2.2. Nucleic Acid Manipulation and Transformation

Primers and plasmids used in this work are listed in Table 2 and Table 3, respectively. Total DNA from *T. thermophilus* was purified using a DNeasy Blood and Tissue kit (Qiagen), following a bacterial culture-adapted protocol. Plasmid construction, purification, restriction analysis, and DNA sequencing were performed by standard methods [35]. DNA was amplified by polymerase chain reaction (PCR) using 1 U mL^−1^ of DNA polymerase from *Pyrococcus furiosus* (Roche Molecular Biochemicals, Basel, Switzerland) in its recommended buffer with 3 mM MgCl_2_, 0.5 mM dNTP mixture, and 50 pmol of each primer (Sigma-Aldrich, St. Louis, MO, USA).

*E. coli* competence was induced following Inoue’s method [36], and transformation was carried out following the method described by Hanahan [33]. Transformation of *T. thermophilus* with linear or circular DNA was achieved by natural competence as described [37].

### 2.3. Construction of Deletion Mutants

Plasmid pUC19 [38] was used for the construction Δ*dnrT*, Δ*dnrS*, and Δ*nsrRST* mutants in *T. thermophilus* through double recombination. For this, 500 bp regions upstream and downstream of each target gene were amplified by PCR with the appropriate primers (Table 2), and cloned into pUC19 using the restriction sites included in the primers. A gene cassette devoid of transcription terminator encoding thermostable resistance to kanamycin (*kat*) [41] was inserted between the cloned fragments using the *XbaI* restriction site incorporated in each construct. Downstream orientation of the resistance cassette was selected, in all cases, to allow the expression of downstream genes. Insertional mutants were subsequently obtained by transformation of *T. thermophilus* with linearized derivatives of each constructs, followed by selection on TB plates with kanamycin (30 mg L^−1^); mutations were confirmed by PCR and, when possible, by western blot.

Complementation assays were carried out with plasmid pWUR, in which cloned genes are expressed under the control of the constitutive promoter *PslpA* (*S*-layer promoter) and with thermostable resistance to bleomycin (15 mg L^−1^).

### 2.4. Promoter Activity Assays

The putative promoter regions that include the 300–500 bp preceding the start codon of the *nsrR* and *nsrS* genes were cloned in the promoter probe plasmid pMHbgaA [30]. Expression from these promoters was assayed on transformed *T. thermophilus* strains in different growth conditions by measuring thermostable *β*-galactosidase activity. For anaerobic expression, cells were grown aerobically up to exponential phase and then subjected to anaerobic conditions for 16 h in the absence or presence of 20 mM nitrate, 5 mM nitrite, or 100 μM SNP.

*β*-Galactosidase activity was assayed twice in triplicate experiments on the chromogenic substrate ortho-nitrophenyl-galactopyranoside (ONPG), (Sigma) at 60 °C. Briefly, cells were permeabilized with 0.2% (*w/v*) SDS for 15 min at 37 °C, followed by addition of the reaction buffer (phosphate 80 mM pH 7.5, containing 0.2% (*w/v*) ONPG). The reaction was incubated for 20 min at 60 °C in a plate reader, and the absorbance variation was measured at 410 nm. *β*-Galactosidase activity was expressed as nanomoles of *o*-nitrophenol produced per min and mg of protein.

### 2.5. Directed Mutagenesis

QuikChange Lightning Site-Directed Mutagenesis Kit (Agilent Technologies, Santa Clara, CA, USA) was used for the construction of the mutant NsrR^C93A^ following manufacturer's instructions. pET22 nsrR plasmid was used as template for the construction of the mutant. Primers used are described in Table 2. The sequence of the mutant was confirmed.

### 2.6. Recombinant Protein Overexpression and Purification

The genes encoding transcriptional regulators *dnrT*, *nsrR*, *nsrS*, and *nsrT* were cloned into pET22b or pET28b vectors (Novagen, Merck KGaA, Darmstadt, Germany) for expression in *E. coli* BL21(DE3) cells, with a His-tag fused to the *N*- or *C*-terminus. Expression was induced at OD_600_ = 0.4 with 1 mM isopropyl *β*-D-1-thiogalactopyranoside (IPTG) for 3 h, after which cell pellets were collected and disrupted by French press. His-tagged proteins were purified by affinity chromatography from the soluble cell fraction on TALON CellThru Resin columns following the manufacturer’s instructions (Clontech Laboratories, Inc., Mountain View, CA, USA). Purified proteins were eluted in elution buffer (50 mM phosphate buffer pH7.0, 300 mM NaCl, 150 mM imidazol) and further dialyzed and concentrated in 50 mM phosphate buffer (pH 7.0) using Amicon Ultra concentrator tubes (3 or 10 kDa cutoff) (Millipore, Burlington, MA, USA). Proteins were visualized by sodium dodecyl sulfate polyacrylamide gel electrophoresis (SDS-PAGE), and concentrations were determined using the Bio-Rad protein assay (Bio-Rad, Hercules, CA, USA) following the manufacturer’s instructions.

Spectrophotometric analysis of the samples was carried out at room temperature in 0.2 mL cuvettes in a V-730 spectrophotometer (Jasco, Oklahoma City, OK, USA).

### 2.7. Western Blot

NarG (α-subunit of the Nar), NirS (*cd*_1_ type Nir), and NorC (*c* subunit of the Nor) were visualized in total cell extracts from nitrate- or nitrite-induced cultures by western blot with specific rabbit antisera [23,32] and horseradish peroxidase-labeled anti-rabbit antibodies; detection was carried out through a electroluminescense (ECL), (Amersham International, Little Chalfont, UK).

### 2.8. Electrophoretic Mobility Shift Assays

Labeled promoter probes were obtained by PCR amplification of the promoter sequences. A 5′-DY782-labeled reverse oligonucleotide (Eurofins MWG Operon, Louisville, KY, USA) was used in the experiments of Figure 5, Figure S5 and S6. PCR products were separated in agarose gels and bands were purified and concentrated using commercial kits (Qiagen, Hilden, Germany).

Interaction reactions were performed in interaction buffer (20 mM 4-(2-hydroxyethyl)-1-piperazineethanesulfonic acid (HEPES) pH 6.9, 50 mM NaCl 50 mM, BSA 0.1 mg mL^−1^, 5 mM *β*-mercaptoethanol) using 25–50 nM labeled DNA and 1:5–1:10 molar ratio of purified transcriptional regulators; 0.5 μg poly(dI–dC) was added as a competitor to reduce non-specific interactions. Reactions were performed for 10 min at 60 °C, after which 2 μL glycerol 85% (*v/v*) was added for gel loading. Six percent EMSA polyacrylamide gels (composition for 10 mL:1 mL 10× EMSA buffer (Tris-Acetate-EDTA (TAE) 50×, 0.5 M EDTA, 0.27% (*v/v*) acetic acid, pH 7.5), 1.5 mL acrylamide/bisacrylamide 40% (37.5:1), 250 μL 1% (*w/v*) APS, 20 μL 100% (*v/v*) tetramethylethylenediamine (TEMED) and water up to 10 mL) were prepared using 1.5 mM glass separators.

Prior to sample loading, gels were pre-run for 20 min at 5 mA in 1× EMSA buffer (see composition above). Electrophoresis was performed at room temperature, protected from the light at 5 mA per gel for approximately 30 min for smaller fragments (100 bp), and up to 3 h for larger fragments (400 bp). Gels were scanned using a LI-COR Odyssey Scanner (LI-COR Biosciences, Lincoln, NE, USA) (excitation 782 nm and emission 800 nm), and analyzed with the Odyssey Infrared Imaging System v.3.0.21 software (LI-COR Biosciences, Lincoln, NE, USA) for those DNA labeled with the DY782 fluorochrome. For unlabeled DNA fragments, staining with SYBR Gold gel staining reagent (Invitrogen, Carlsbad, CA, USA) was used, and the DNA detected at 300 nm.

## 3. Results

### 3.1. Genes and Putative Promoters of the Nic Cluster

**Figure 1:**
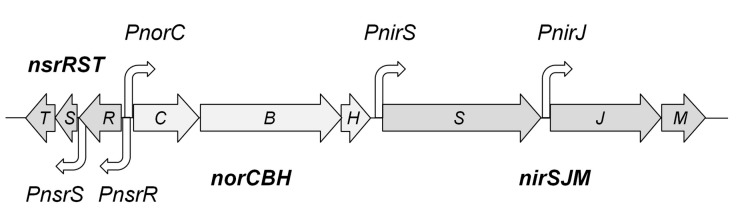
The nitrate and nitric oxide respiration cluster (*nic*). The nitrite reductase operon includes the NirS *cd*_1_ nitrite reductase, the NirJ maturation protein and the NirM cytochrome *c*. NO reductase is formed by a complex including NorC cytochrome *c*, NorB NO reductase, and NorH; promoters *PnirS*, *PnirJ*, and *PnorC* have been previously detected, and putative promoters *PnsrR* and *PnsrS* are indicated.

In previous studies, we have shown that the genes for nitrite and NO respiration in *T. thermophilus* PRQ25 are expressed from the *PnirS*, *PnirJ*, and *PnorC* promoters [28,29] (Figure 1). In addition, the *nic* cluster encodes, in opposite orientation to the *norC* gene, three small proteins of 141 aa, 65 aa, and 98 aa (Accession number: FN666415), which are highly conserved in sequence and organization in the *nic* clusters from all the denitrifying *Thermus* spp. sequenced so far [24]. The 141 amino acid (aa) protein is predicted to be cytoplasmic, and its sequence identifies it as a member of the BadM/Rrf2 family of transcriptional regulators, similar in sequence to NsrR proteins involved in detection of NO in other microorganisms [42,43,44,45,46]. We will refer to this protein as NsrR^Th^ in this work, for clarity. Similar to its homologues, the NsrR^Th^ protein sequence includes an *N*-terminal HTH DNA-binding domain, and three conserved cysteines in its *C*-terminal domain, which in other bacteria, coordinate a “sensory” iron–sulfur cluster (Supplementary Figure S1). However, the glutamic acid that constitutes the fourth iron–sulfur cluster ligand in NsrR proteins [13] seems to be replaced by serine (S85) in NsrR^Th^.

The second protein in the cluster, NsrS, has also a predicted cytoplasmic localization and contains a ScdA domain (PF04405) commonly present in repair of iron clusters (RIC) proteins [47]. Finally, the last protein in this cluster, NsrT, is predicted to contain a Cupin 2 domain, a small conserved barrel domain of unknown function, found in diverse proteins.

The putative promoter responsible for the expression of *nsrR* is likely located within the 129 bp region that separates its coding sequence from that of the divergent *norC* gene (Figure 1). The 47 bp that separate the *nsrR* stop codon from the start codon of *nsrS* is much longer than the usual distance between co-transcribed genes in the compact genome of *T. thermophilus*, suggesting the existence of a specific promoter that controls the expression of the NsrS–NsrT proteins. In contrast, the stop codon of *nsrS* overlaps the start codon of *nsrT*, strongly supporting *nrsS–nrsT* co-transcription. However, transcripts spanning *nsrR*, *nsrS*, and *nsrT* were undetectable by real-time reverse transcriptase polymerase chain reaction (RT-PCR) (data not shown), suggesting that the three genes are transcribed at very low levels, even under denitrification conditions. To check this, we tested the expression of a thermostable *β*-galactosidase reporter from the *PnsrR* and *PnsrS* putative promoters in a multicopy promoter probe plasmid, both in the aerobic strain HB27 and its denitrifying derivative HB27d. Only the putative *PnsrS* promoter showed a two-fold increase in expression above the background in the HB27d strain under anoxia plus nitrate growth conditions (Supplementary Figure S2). Low expression levels from the putative *PnsrR* promoter were confirmed in these assays, which demonstrated the presence of an independent promoter that controls the expression of a *nsrST* transcript.

### 3.2. Regulation of the Nitrite and NO Reductases Gene Promoters

**Figure 2:**
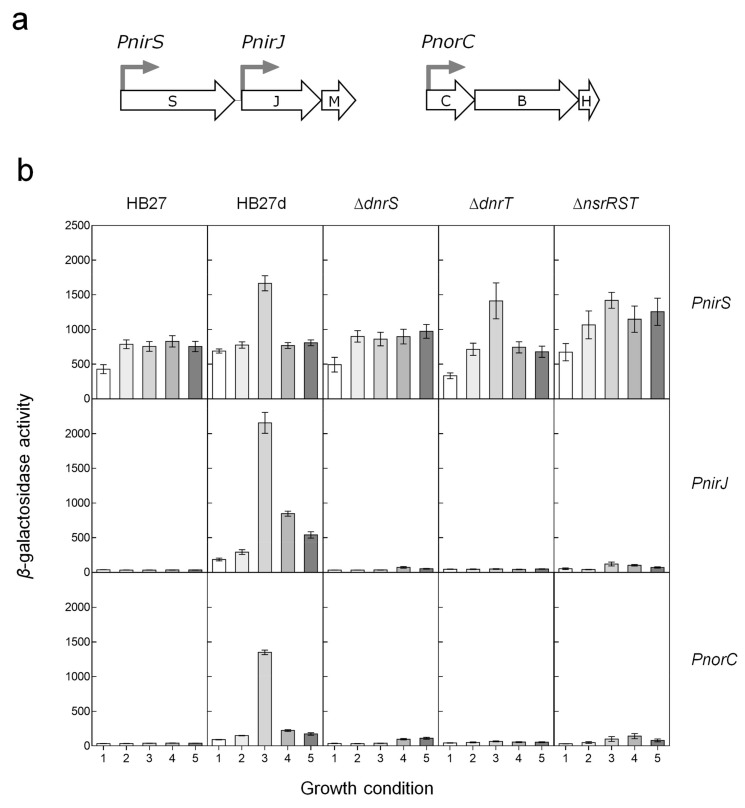
Transcriptional activity from the *nic* promoters. (**a**) Scheme of the promoters assayed; (**b**) *β*-galactosidase activity of the strains HB27, HB27d, HB27d *dnrS::kat* (∆*dnrS*), *HB27d dnrT::kat* (∆*dnrT*), and HB27d *nsrRST::kat* (∆*nsrRST*) carrying the promoter probe plasmids, pMHPnirSbgaA (*PnirS*), pMHPnirJbga (*PnirJ*) or pMHPnorCbgaA (*PnorC*). Transcriptional activity was measured in aerobic cultures (1) or after induction for 16 h under anaerobic conditions in the absence (2) or presence of 20 mM nitrate (3); 5 mM nitrite (4); or 100 μM SNP (5). *β*-Galactosidase activity is expressed as nanomoles of *o*-nitrophenol produced per min and mg of protein. Data represent mean values from triplicate samples in at least two independent experiments; bars indicate standard error.

**Figure 3:**
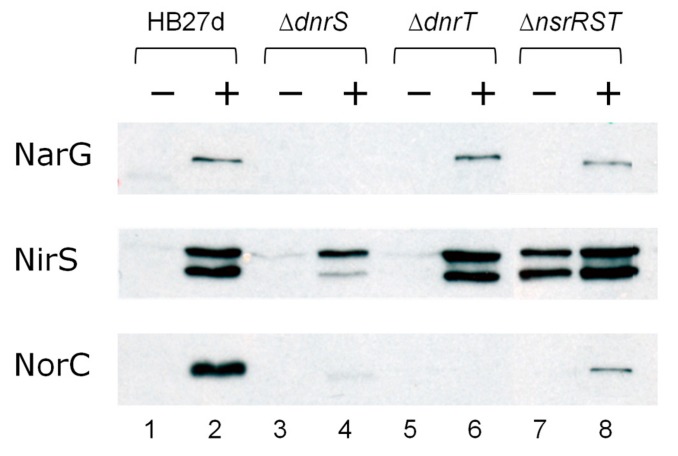
Expression of denitrification reductases in the regulator mutants. Immunodetection of NarG (α-subunit of the Nar, 136 kDa), NirS (*cd*_1_ type Nir, 57 and 48 kDa), and NorC (c subunit of the Nor, 25 kDa) after 16 h under anaerobic conditions in the absence (−) or presence of 20 mM nitrate (+) as electron acceptor. Strains: HB27d, HB27d *dnrS::kat* (∆*dnrS*), HB27d *dnrT::kat* (∆*dnrT*), and HB27d *nsrRST::kat* (∆*nsrRST*).

The low expression levels from the *PnsrR* and *PnsrS* promoters precluded their further analysis; in contrast, the *PnorC*, *PnirS*, and *PnirJ* promoters showed high expression levels using the same promoter probe vectors (Figure 2). Expression from *PnirS* was almost constitutive in the aerobic strain HB27, and its expression was increased two-fold by nitrate under anoxic conditions in HB27d, consistent with previous observations [28]. Deletion mutants were used to analyze the roles of the NsrR^Th^, NsrS, and NsrT proteins, and the DnrS and DnrT regulators in Nir transcription. Nitrate-mediated anaerobic induction was not detected in the absence of DnrS, whereas absence of DnrT had almost no effect on *PnirS* expression (Figure 2). On the other hand, deletion of the *nsrRST* cluster produced around two-fold upregulation of *PnirS*, especially under anoxic conditions, which was confirmed at the protein level by Western blot tests against NirS (Figure 3). In this figure, the holo (larger) and apo (smaller) forms of NirS were clearly detected in the Δ*nsrRST* mutant grown in the absence of nitrate (Figure 3, lane 7), in contrast to the negligible detection in the parental strain under the same conditions (Figure 3, lane 1). Further, the absence of induction in *PnirS* transcription of the Δ*dnrS* mutant upon growth with nitrate observed in Figure 2 was confirmed by Western blot (Figure 3, lane 4 vs. lane 2), as was the lack of effect of Δ*dnrT* mutation (Figure 3, lane 6 vs. lane 2). Parallel detection of the nitrate reductase (NarG) encoded within the NCE confirmed its requirement for DnrS but not for DnrT [30], and also independence of NsrR^Th^ (Figure 3, lanes 2, 4, 6, and 8).

On the other hand, both the *PnirJ* and the *PnorC* promoters behaved in a similar way to each other. As expected, these promoters were not active in the strictly aerobic HB27 strain (Figure 2). However, both were induced by anoxia and nitrate in the denitrifying HB27d strain, consistent with the requirement for specific transcriptional activators encoded within the denitrification clusters. Other nitrogen oxides, such as nitrite or NO (produced from SNP), also led to an increased transcriptional activity, but with lower efficiency than nitrate. For the *PnirJ* and the *PnorC* promoters, absence of the DnrS, DnrT, or *nsrRST* cluster led to complete loss of transcription under all the conditions assayed (Figure 2). Detection of NorC by Western blot confirmed the absence of NorC expression in Δ*dnrT* mutants (Figure 3, lane 6), and very low expression in Δ*dnrS* and Δ*nsrRST* (Figure 3, lanes 4 and 8) mutants, compared to the wild type parental strain. Expression from *PnirJ* could not be confirmed by Western blot, due to the lack of an available NirJ-specific antibody.

In conclusion, while induction of NirS by nitrate depended only partially on DnrS and was repressed by protein(s) expressed from the *nsrRST* cluster, expression of NorC and NirJ required both DnrS and DnrT, and at least one of the proteins encoded by the *nsrRST* cluster, likely NsrR^Th^.

### 3.3. NsrR^Th^ is a Transcription Factor

**Figure 4:**
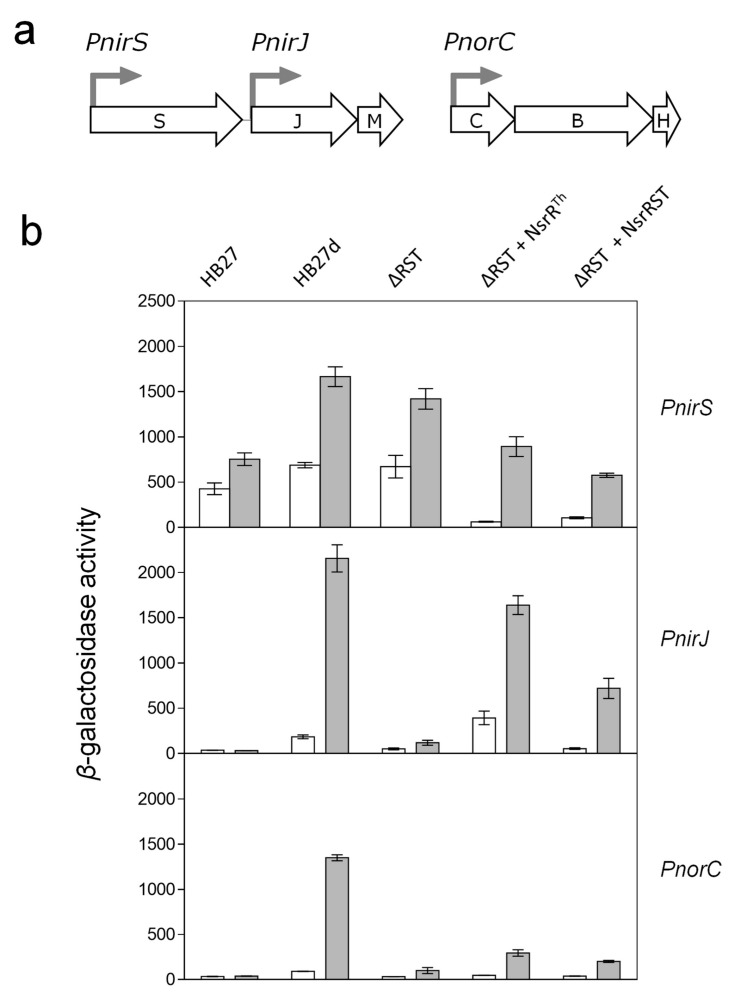
Complementation of promoter expression by NsrR^Th^. Transcriptional activity of promoters *PnirS*, *PnirJ*, and *PnorC* (positions indicated in (**a**)) in HB27, HB27d and HB27d ∆*nsrRST::kat* (∆RST) strains complemented with plasmids overexpressing NsrR^Th^ (∆RST + NsrR), or all three proteins (∆RST + NsrRST) (**b**). Transcriptional activity was measured in aerobic cultures (white) or after induction for 16 h under anaerobic conditions with 20 mM nitrate (grey). *β*-Galactosidase activity is expressed as nanomoles of *o*-nitrophenol produced per min and mg of protein. Data represent mean values from triplicate samples in at least two independent experiments; bars indicate standard error.

To elucidate the role of NsrR^Th^ in the expression of the *nic* promoters Δ*nsrRST* complementation studies were carried out. For this purpose, the *nsrR* gene and the whole *nsrRST* cluster were expressed from plasmids pWURnsrR and pWURnsrRST, derived from pWUR112/77-1 [40], a bifunctional *E. coli–Thermus* vector conferring thermostable bleomycin resistance that permits medium-to-high levels of constitutive expression of the cloned genes. Each plasmid was co-transformed with the promoter probe vectors into the Δ*nsrRST* mutant, and thermostable *β*-galactosidase activity was measured under aerobic and anaerobic conditions. As shown in Figure 4, the plasmid expressing NsrR^Th^ produced strong activation of *PnirJ* and partial activation on *PnorC*, whereas *PnirS* was partially repressed. The collective ectopic expression of all three genes (*nsrR*, *nsrS* and *nsrT*) produced a similar effect as NsrR^Th^ alone, although reaching lower activity levels.

### 3.4. NsrR^Th^ Binds to the Main Nic Promoters

**Figure 5:**
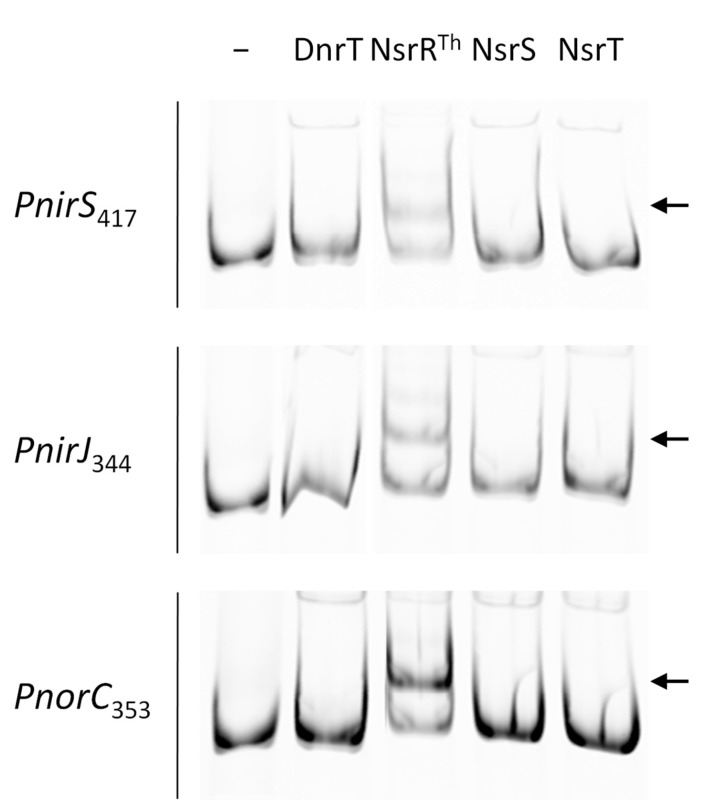
NsrR^Th^ binding to the *nic* promoters. Promoters *PnirS*, *PnirJ*, and *PnorC* labeled with DY782 were used as probes in electrophoretic mobility shift assays (EMSA) with the indicated purified proteins. Arrows indicate the mobility of specific DNA–protein complexes. Promoter probes (50 nM) were incubated in interaction buffer with each regulator (500 nM) at a 1:10 ratio for 10 min at 60 °C. Lane (−) corresponds to control without protein.

The above results support the notion that NsrR^Th^ can function as a transcriptional activator for the *PnirJ* and *PnorC* promoters, and as repressor of *PnirS* promoter. To determine the ability of NsrR^Th^ to bind DNA, His-tagged NsrR^Th^, NsrS, NsrT, and DnrT proteins were expressed and purified by affinity chromatography; products of expected size were obtained, though NsrT seemed to produce SDS-resistant dimers (Supplementary Figure S3). With these proteins, EMSA assays were carried out using DNA fragments of 417, 344, and 353 base pairs (bp) containing the *PnirS*, *PnirJ*, and *PnorC* putative promoters, respectively. Significant DNA–protein complexes were detected for NsrR^Th^ in all three promoters, whereas none of the other proteins, including DnrT, had any detectable effect on DNA mobility (Figure 5).

As NsrS and NsrT are always encoded in tandem with NsrR^Th^ in diverse *Thermus* spp., we searched for the effects of NsrS and NsrT on NsrR^Th^’s DNA binding. Addition of each protein alone, or in combination, did not affect the binding capacity of NsrR^Th^ to the *PnorC* promoter under the conditions assayed (Supplementary Figure S5).

### 3.5. Identification of the Putative Binding Sites of NsrR^Th^

**Figure 6:**
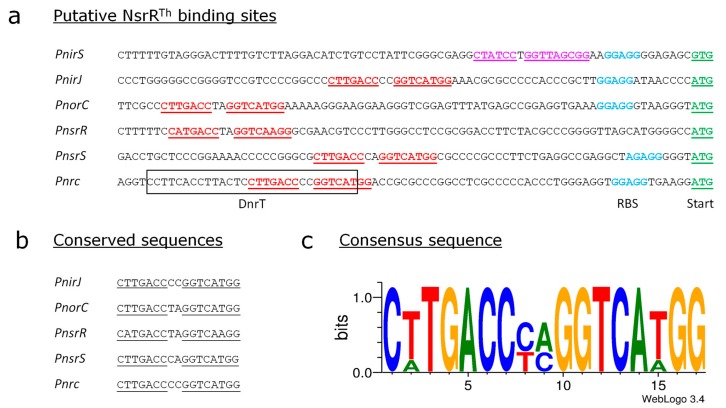
Sequence analysis of the *nic* promoters. (**a**) The ribosome binding sites (RBS) and the ATG codons of the indicated genes are highlighted in blue and green, respectively. Conserved sequences found in all promoters are underlined and highlighted in red. The DnrT binding site in *nrc_p_* is framed in a black box; (**b**) Conserved putative NsrR^Th^ binding sites; (**c**) WebLogo [48,49] generated by aligning the putative NsrR^Th^ binding sites, excluding the one found in *PnirS*.

A sequence analysis revealed the presence of a highly conserved palindromic sequence “CTTGACCNNGGTCATGG” in those promoters regulated by NsrR^Th^ (Figure 6). This sequence partially overlaps with the binding fingerprint generated by DnrT on the *Pnrc* promoter [30] (framed in Figure 6a). This sequence localizes upstream of the translation start (−50 for the *PnirJ* promoter, −73 for the *PnorC* promoter, −61 for the *Pnrc* promoter), except for the *PnirS* promoter, in which it is localized immediately before the identified Shine–Dalgarno sequence (−30). The proximity to the translation start of NirS, and the lower sequence conservation, points out to a repressive effect of NsrR^Th^ on the *nirS* expression compared to the activation that takes place on the *nirJM* and the *norCBH* operons, in agreement with our previous results. Both the *PnsrR* and the *PnsrS* promoter regions contain this conserved sequence “CTTGACCNNGGTCATGG” at a similar distance from the ATG start codon (−72 and −52, respectively) found in the *PnirJ* promoter, supporting that the *nsrRST* genes are also regulated by NsrR^Th^.

To confirm the requirement of this sequence for the binding of NsrR^Th^ to the promoter sequences, we performed EMSA assays with *PnorC* fragments of different length, with or without the putative NsrR^Th^ binding site (Supplementary Figure S6a). Our results show that addition of NsrR^Th^ did not cause a shift when *PnorC* fragments lacking the palindromic sequence were used (Supplementary Figure S6b, fragments −56 and −51), thus confirming that NsrR^Th^ binds to this conserved sequence.

## 4. Discussion

In this article, we show that the expression of the nitrite reductase and the nitric oxide reductases of the denitrification pathway of *T. thermophilus* depends on the oxygen sensitive global regulator DnrS and on the local regulator NsrR^Th^, which in contrast to homologues in other bacteria, functions both as repressor and as activator of specific denitrification promoters.

### 4.1. DnrS and DnrT as Master Regulators of Denitrification.

In nitrate-respiring isolates of *T. thermophilus*, the master regulators DnrS and DnrT are required for the expression of the nitrate reductase, and consequently, deletion mutants in either of these proteins prevented anaerobic growth with nitrate [30]. In denitrifying isolates of the same species, the deletion of these regulators also prevents anaerobic growth with nitrite and NO [22], supporting that both proteins are involved in the control of the expression of the genes encoding the nitrite and the nitric oxide reductases in their natural host. Here, we analyze this hierarchy of control in a formerly aerobic subrogate host that has received the adaptive NCE and *nic* clusters by consecutive transformation events [23]. In this host, de novo adapted to an anaerobic lifestyle, we demonstrate the role of these NCE-encoded transcription factors as master regulators of the promoters in the *nic* cluster, thus showing the hierarchical dominance of the NCE over the *nic* cluster. Ultimately, we show that the transcription factor NsrR^Th^ regulates the *nic* cluster subordinated to the NCE master regulators.

Under aerobic conditions, the nitrite reductase gene is transcribed from the *PnirS* promoter at basal levels, whereas anaerobic incubation in nitrate-containing media produces a DnrS-dependent two-fold induction (Figure 2). In contrast, DnrT seems to play no role in NirS expression (Figure 2 and Figure 3). In addition, our data support the involvement of a transcriptional repressor encoded within the *nsrRST* cluster in the control of *PnirS*, as mutants lacking this cluster show upregulation of *PnirS* transcription and NirS expression under anoxia, independently of the presence or absence of nitrogen oxides (Figure 2 and Figure 3).

In contrast, transcription from the *PnirJ* and *PnorC* promoters is not induced at all by anoxia, except when nitrogen oxides are present (nitrate > nitrite > NO). Such anoxia plus nitrogen oxide activation requires both DnrS and DnrT, and at least one of the proteins encoded by the *nsrRST* cluster (Figure 2). Their similar behavior suggests that both *PnirJ* and *PnorC* promoters are co-regulated in an analogous way, with at least three transcription activators working on them. However, a more simplistic alternative could involve a dependence effect between these putative activators affecting their own expression. Actually, DnrS mutants (Δ*dnrS::kat*) express DnrT in a constitutive form, and therefore, absence of transcription from *PnorC* and *PnirJ* promoters implies a direct effect of DnrS. In contrast, DnrT mutants (Δ*dnrT::kat*) do not express DnrS at all [30], making it impossible to discriminate if the detected effects on *PnorC* and *PnirJ* are due to DnrT or to DnrS absence. In any case, both promoters contain sequences that partially overlap with the binding fingerprint generated by DnrT on the *Pnrc* promoter [30] (framed in Figure 6), supporting the existence of a direct binding of DnrT also on *PnorC* and *PnirJ*.

### 4.2. The Role of NsrR^Th^

The conserved *nsrRST* cluster is proximal to the Nir and Nor reductase genes in different *Thermus* spp., such as *T. thermophilus* SG0.5JP17-16 and *T. scotoductus* SA01. Mutants of the HB27d strain lacking the *nsrRST* cluster used in this work were unable to grow anaerobically with nitrite (data not shown), consistent with the lack of transcription from the *PnorC* and *PnirJ* promoters. Of the three hypothetical proteins encoded in the cluster, only NsrR^Th^ shows a canonical DNA binding motif and in vitro binding capability to the *PnirS*, *PnirJ*, and *PnorC* promoters in EMSA assays (Figure 5). In addition to this DNA binding motif, NsrR^Th^ contains three cysteines conserved in NsrR homologs from other bacteria, where they coordinate a [4Fe–4S] iron–sulfur cluster that functions as NO sensor [13], with a conserved fourth ligand absent in NsrR^Th^. The presence of an iron–sulfur cluster in NsrR^Th^ has been demonstrated (Figure S4), suggesting that another glutamic residue in the vicinity (i.e., E82) could constitute the fourth ligand for its iron–sulfur cluster.

Our promoter assays suggest that, under denitrification conditions, NsrR^Th^ represses *PnirS*, whereas it induces *PnirJ* and *PnorC* (Figure 4). Our EMSA assays actually show binding capability of NsrR^Th^ to all the *nic* promoters. It remains to be analyzed whether the dual activator/repressor role of NsrR^Th^ can be modulated by the oxidation/reduction state of its iron–sulfur cluster. Since EMSA assays were carried out under aerobic conditions, it is likely that the NsrR^Th^ recombinant protein used was actually a partially oxidized form of the protein, already able to bind DNA.

Another important factor concerning the regulation of these promoters is the location of the putative NsrR^Th^ binding site, because most probably it can determine the outcome of the regulation in each specific promoter context, that being induction of the *PnirJ* and *PnorC* promoters, or repression of the *PnirS* promoter (Figure 6). The localization of this sequence, close to the translation start in the *PnirS* promoter and further upstream in the *PnirJ* and *PnorC* promoters, agrees with the role of NsrR^Th^ as repressor of *PnirS* and activator of *PnirJ* and *PnorC*, deduced from our in vivo assays.

### 4.3. The Role of NsrS and NsrT

The *PnsrS* promoter region contains the conserved sequence “CTTGACCNNGGTCATGG” at a similar distance from the ATG start codon found in the *PnirJ* and *PnorC* promoters, supporting that the NsrS and NsrT proteins are also regulated by NsrR^Th^. However, the role of these proteins remains to be elucidated. There is no doubt about their involvement in denitrification both from their sequence conservation and from their clustering downstream of *nsrR* homologues in denitrifying *Thermus* spp. A search for homologues in GenBank revealed a ScdA domain (PF04405) within NsrS, taking its name from the protein ScdA of *Staphylococcus aureus*, also present in proteins YftE of *E. coli* and DnrN of *Neisseria gonorrhoeae*, which are both described as di-iron proteins involved in the repair of iron–sulfur clusters or repair of iron centers (RIC) [47]. Mutations in these RIC proteins in model organisms produce pleiotropic effects when subjected to NO and other oxidative stresses. Interestingly, RIC proteins are upregulated by NO [50], and their coding genes are usually located immediately upstream or downstream of regulatory proteins of the NsrR family in denitrifying bacteria, such as *Ralstonia eutropha*, *Ralstonia solanacearum*, *Hahella chejuensis*, and *Anaeromyxobacter dehalogenans*, supporting a similar role for all of them. In fact, a detailed analysis of NsrS showed sequence homology with the *N*-terminal region of RIC proteins, including a highly conserved DfCCgG motif of unknown function [50]. However, most RIC proteins are much larger (around 220 aa) than NsrS (65 aa), and their *C*-terminal domains are involved in binding of a non-heme bi-nuclear iron center. We hypothesize that the cupin-2 domain of the NsrT protein, which also contains two cysteine residues, could play a role in iron binding, similar to that of the *C*-terminal domain of the RIC proteins. Overall, these data suggest a joint role for the NsrS and NsrT proteins, likely co-translated in 1:1 stoichiometry, in the repair of the iron–sulfur center of NsrR^Th^, in a similar way to the role played by the RIC proteins of other bacteria. In any case, further investigations are required to determine the specific role of NsrS and NsrT in denitrification.

### 4.4. A Regulatory Model for Nitrite Respiration

**Figure 7:**
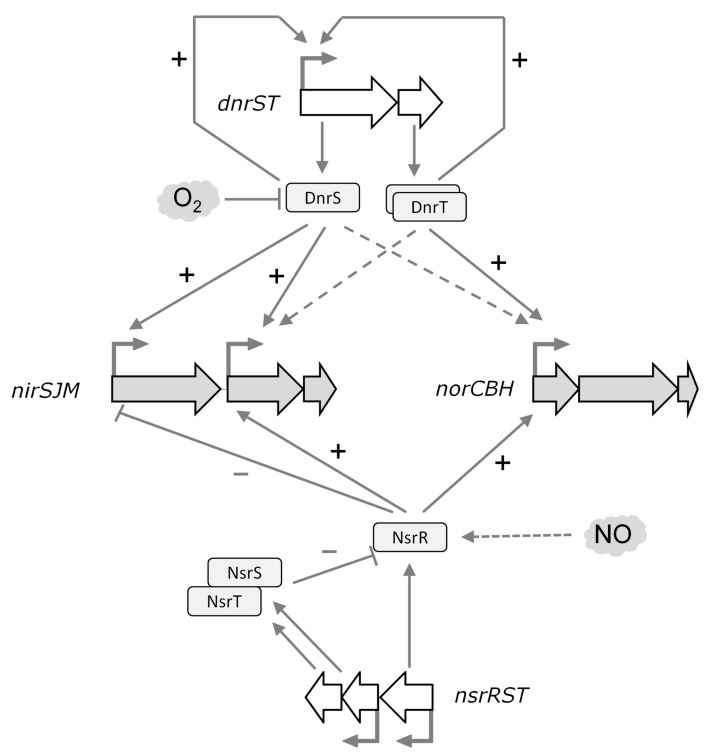
The regulatory network of the *nic* cluster. DnrS and DnrT encoded by the nitrate respiration cluster play a major role in the control of the denitrification pathway, being required for transcription of nitric oxide (NO) reductase and also of the *nirJ* gene. The transcription of *nirS* is semi-constitutive except for a two-fold enhancement that depends on DnrS but not on DnrT. Activation (+) and repression (−) are indicated. Dashed arrows indicate a putative indirect effect of DnrT and DnrS. NsrR^Th^ is a transcription factor required for the expression of the *nic* operons. NsrR^Th^ binds to the regulated promoters, activating NO reduction by Nor and limiting its production by NirS. The putative activity of NsrS and NsrT in the repair of the iron–sulfur cluster of NsrR^Th^ remains to be elucidated.

A complex regulatory network of the *nic* cluster can be depicted in *T. thermophilus* (Figure 7). In natural denitrifying strains, the denitrification reductases initiate a cascade of reactions that lead to the subsequent reduction of nitrate, nitrite, and NO, leading to the final production of N_2_O. In this process, reduction of nitrite to NO has to be immediately followed by further reduction to N_2_O, given the toxic effects of NO accumulation. Therefore, a tight regulation is required. As a first control level, the *nic* cluster cannot be expressed in the absence of the NCE, because DnrS and DnrT are master regulators required to pre-activate the nitrite respiration promoters upon oxygen depletion. However, transcription of *nirS* is semi-constitutive, except for a two-fold enhancement that depends on DnrS, but not on DnrT. This fact could lead to the production of NO up to toxic levels, even in strains that only have the *nic* cluster by lateral gene transfer, so preventive repressor systems should be in place. According to our model, NsrR^Th^ could constitute such a security mechanism in which reaction with NO excess allows binding to the *PnirS* promoter with subsequent reduction in NO production by Nir. Concomitantly, NsrR^Th^ would also activate the production of Nor through binding to its gene promoter and decreasing the NO levels in the cell. Finally, the other two proteins encoded along with NsrR^Th^, NsrS and NsrT, could play a key role in restoring the activity of the NsrR^Th^ iron–sulfur cluster, returning the system to its basal level.

## Figures and Tables

**Table 1:** Strains used in this work.

Name	Genotype/Phenotype	Reference
DH5α	F- *endA1 glnV44 thi-1 recA1 relA1 gyrA96 deoR nupG* Φ80 *lacZ*ΔM15 Δ(*lacZYA-argF*)U169, *hsdR*17 (rK- mK+), λ-	[33]
BL21 (DE3)	F- *ompT gal dcm lon hsdSB* (rB- mB-) λ(DE3 [*lacI lacUV5*-T7 gene 1 *ind*1 *sam7 nin5*])	[34]
*T. thermophilus* HB27	Wild type	Dr. Koyama
*T. thermophilus* HB27d	Denitrifying strain	[23]
HB27d ∆*dnrS::kat*	*dnrS* mutant	This work
HB27d ∆*dnrT::kat*	*dnrT* mutant	This work
HB27d ∆*nsrRST::kat*	*nsrRST* mutant	This work

**Table 2:** Oligonucleotides used in this work. Underlined sequences correspond to restriction sites included for cloning purposes.

Name	Sequence	Amplicon/Purpose
PnsrRXbaIdir	AAAATCTAGAGGAGTCCACCGTCAGGT	*PnsrR*/promoter probe plasmid
PnsrRNdeIrev	AAAACATATGCCCATGCTAACCCCGG	*PnsrR*/promoter probe plasmid
PnsrSXbaIdir	AAAATCTAGAGTGGAAAGCCGCGTGG	*PnsrS*/promoter probe plasmid
PnsrSNdeIrev	AAAACATATGCCCTCTAGCCTCGGC	*PnsrS*/promoter probe plasmid
katXbaIdir	AAAATCTAGACCCGGGAGTATAACAGA	*kat*/resistance cassette
katXbaIrev	AAAATCTAGACGTTCAAAATGGTATGCGTTTTGA	*kat*/resistance cassette
MutSa	AAAAGAATTCGCGTTTGGCGGCGTC	*dnrS*/deletion mutant
MutSb	AAAATCTAGAGCGAGGGCCTCCC	*dnrS*/deletion mutant
MutSc	AAAATCTAGAGGGGGTGAGGCCGT	*dnrS*/deletion mutant
MutSd	AAAAAAGCTTGGCGTGGCAGCGG	*dnrS*/deletion mutant
MutTa	AAAAGAATTCACCGCCCTGCGGC	*dnrT*/deletion mutant
MutTb	AAAATCTAGACTCCACGGCCTCACC	*dnrT*/deletion mutant
MutTc	AAATTCTAGATAAACGCGAGCGGTTCT	*dnrT*/deletion mutant
MutTd	AAAAAAGCTTCGCGCCTCCTCGG	*dnrT*/deletion mutant
nsrR 3’ dir	AAAATCTAGAGGCCCCATGCTAACCCCG	*nsrRST*/deletion mutant
nsrR 3’ rev	AAAAGTCGACTTGCGGCTCTGCAGGGTCAT	*nsrRST*/deletion mutant
nsrT 5’ dir	AAAAGAATTCAGGGGCTCGAGGGTGAAC	*nsrRST*/deletion mutant
nsrT 5’ rev	AAAATCTAGATGATCCCCGCAAGCCGCC	*nsrRST*/deletion mutant
nsrRrbsXbadir	AAAATCTAGAGGAGGATAGATGGCCCTTCGGAGC	*nsrR*/complementation plasmid
nsrRNdeIdir	AAAACATATGGCCCTTCGGAGCCTTC	*nsrR*/overexpression plasmid
nsrR+stopEcorev	AAATGAATTCTCAAGCGCCCGGGGG	*nsrR*/complementation plasmid
nsrR-stopEcorev	AAAAGAATTCGCGCCCGGGGG	*nsrR*/overexpression plasmid
nsrSNdeIdir2	AAAACATATGGAAGGCCTCACCCTAAG	*nsrS*/overexpression plasmid
nsrS+stopEcorev	AAATGAATTCTCATGCCCTTCCCTCC	*nsrS*/overexpression plasmid
nsrTNdeIdir	AAAACATATGAACCTCTTGGAAAAGGC	*nsrT*/overexpression plasmid
nsrT+stopEcoRIrev	AAAAGAATTCATGGCCTCGGGGTGAT	*nsrT*/overexpression plasmid
dnrTNdeIdir	AAAACATATGGAGCTCGCCCAG	*dnrT*/overexpression plasmid
dnrTEcoRIrev	AATTGAATTCTTAGCGGATCAGGGC	*dnrT*/overexpression plasmid
PnirXbaIdir	AAAATCTAGAGCGCGACCTTATGCTCTACG	*PnirS*/EMSA *PnirS*_417_
PnirDY782_rev	[DY782]AAAACATATGCTCCCCTCCTTCCGCTAAC	*PnirS*/EMSA *PnirS*_417_ (fluorescent label)
Pnir2XbaIdir	AAAATCTAGACTTGAAGTCACCAAGTGCTGG	*PnirJ*/EMSA *PnirJ*_344_
Pnir2DY782_rev	[DY782]AAAACATATGGTTATCCTCCAAGCGGGTGG	*PnirJ*/EMSA *PnirJ*_344_ (fluorescent label)
PnorXbaIdir	AAAATCTAGACTTGGGCCACACCCCTC	*PnorC*/EMSA *PnorC*_353_
PnorDY782_rev	[DY782]AAAACATATGCTTACCCTCCTTTCACCTCCG	*PnorC*/EMSA *PnorC*_353_ (fluorescent label)
Pnor_s -98_dir	CGCGGAGGCCCAAG	EMSA *PnorC* -98
Pnor_s -85_dir	GGGACGTTCGCCCTTGAC	EMSA *PnorC* -85
Pnor_s -75_dir	CCCTTGACCTAG	EMSA *PnorC* -75
Pnor_s -64_dir	GGTCATGGAAAAA	EMSA *PnorC* -64
Pnor -56_dir	AAAAAGGGAAGGAAGGGTCGGAGTTTATGAGCCGGAGGTGAAAGGAGGGTAAG	EMSA *PnorC* -56
Pnor -56_rev	CTTACCCTCCTTTCACCTCCGGCTCATAAACTCCGACCCTTCCTTCCCTTTTT	EMSA *PnorC* -56
Pnor -51_dir	GGAAGGGTCGGAGTTTATGAGCCGGAGGTGAAAGGAGGGTAAG	EMSA *PnorC* -51
Pnor -51_rev	CTTACCCTCCTTTCACCTCCGGCTCATAAACTCCGACCCTTCC	EMSA *PnorC* -51
NsrR C93A Fw	GGACCTCGCCGCCACC	*nsrR*/mutation
NsrR C93A Rv	GGTGGCGGCGAGGTCC	*nsrR*/mutation

**Table 3:** Plasmids used in this work.

Plasmid	Use	Reference
pMHbgaA	Empty promoter probe vector. HygR.	[30]
pMHPnsrRbgaA	*PnsrR* promoter probe vector. HygR.	This work
pMHPnsrSbgaA	*PnsrS* promoter probe vector. HygR.	This work
pUC19	Cloning vector. AmpR	[38]
pUC19 ∆dnrS::kat	*dnrS* mutant construction. AmpR, KanR.	This work
pUC19 ∆dnrT::kat	*dnrT* mutant construction. AmpR, KanR.	This work
pUC19 nsrRST::kat	*nsrRST* mutant construction. AmpR, KanR.	This work
pMHPnirSbgaA	*PnirS* promoter probe vector. HygR.	[28]
pMHPnirJbgaA	*PnirJ* promoter probe vector. HygR.	[28]
pMHPnorCbgaA	*PnorC* promoter probe vector. HygR.	[39]
pWUR112/77-1	Expression vector in *T. thermophilus.*BleoR.	[40]
pWURnsrR	Complementation of NsrR^Th^. BleoR.	This work
pWURnsrRST	Complementation of NsrR^Th^ST. BleoR.	This work
pET22b(+)	Expression vector in *E. coli*. AmpR.	Novagen, Merck KGaA, Darmstadt, Germany
pET28b(+)	Expression vector in *E. coli*. KanR.	Novagen, Merck KGaA, Darmstadt, Germany
pET28 dnrT	Overexpression of His-DnrT. KanR.	This work
pET22 nsrR	Overexpression of NsrR^Th^ -His. AmpR.	This work
pET28 nsrS	Overexpression of His-NsrS. KanR.	This work
pET28 nsrT	Overexpression of His-NsrT. KanR.	This work
pET22 nsrRC93A	Overexpression of NsrR^C93A^-His. AmpR	This work

## Supplementary Materials

The following are available online at www.mdpi.com/2073-4425/8/12/361/s1. Figure S1: Sequence alignment of NsrR^Th^ with NsrR family members; Figure S2: Transcriptional activity from the putative promoters of the *nsrR*, *nsrS* and *nsrT* genes; Figure S3: Production of recombinant His-tagged proteins; Figure S4: Production and spectroscopic analysis of NsrR^Th^ and its C93A mutant; Figure S5: Effects of NsrT and NsrS on the binding of NsrR^Th^ to the *PnorC* promoter; Figure S6: NsrR^Th^ binds to a conserved palindromic sequence.
