# Supplementary material for: Hierarchical Control of Nitrite Respiration by Transcription Factors Encoded within Mobile Gene Clusters of Thermus thermophilus
Source: Genes (Basel). 2017 Dec 1;8(12):361. doi: 10.3390/genes8120361 (PMC5748679; doi:10.3390/genes8120361)
Supplement: Supplementary file 1 [file genes-08-00361-s001.pdf]

Supplementary material

```
NgNsrR       ----MYLTQHTDYGLRVLIYTAVNDD-A-LVNIATIASTYGISKSHLMKVVTALVKGGFL   54
RcNsrR       ----MHLLASTDFALRALLFLATDPE-R-LVNTETMSRDLGISRNHLQKVVQALVAGGFA   54
BsNsrR       ----MKLTNYTDYSLRVLIFLAAERPGE-LSNIKQIAETYSISKNHLMKVIYRLGQLGYV   55
NeNsrR       ----MRLTNYSDYALRILTYLGLKR-EE-LSTITEIADCYGISRNHVVKIVHHLGQLGYV   54
EcNsrR       ----MQLTSFTDYGLRALIYMASLPEGR-MTSISEVTDVYGVSRNHMVKIINQLSRAGYV   55
ScNsrR       ----MRLTKFTDLALRSLMRLAVVRDGDEPLATREVAEVVGVPYTHAAKAITRLQHLGVV   56
GlBadM       ---MMELTRKGDYAIRGIIYLASQPPNK-ISLLSEIAVAVDVPQTFLAKIFQQFSKTGIV   56
TthNsrR      MALRSLLKREESYALHALLLLAEEPGLS----ALEIAERLKAPPAFMAKVLQKLAKAGLV   56
                   *    . .:: :   .             ::        .  * .  :   *

NgNsrR       HSVRGKGGGLRLAAPPERINIGAVVRHLEPM-QLVECMG-PNNECLITP---SCRLTGIL   109
RcNsrR       RTIKGPRGGVRLAHPATEIRIGAVVRHFEEHQPIVACFA-PEGQCVIEP---ICGLKGVL   110
BsNsrR       ETIRGRGGGIRLGMDPEDINIGEVVRKTEDDFNIVECFDVNKNLCVISP---VCGLKHVL   112
NeNsrR       DTLRGKNGGIRLAHAPEKINIGEVIRHTETSMDIVECFS-NQNSCIIGC---SCVLRTAI   110
EcNsrR       TAVRGKNGGIRLGKPASAIRIGDVVRELEPL-SLVNCS---SEFCHITP---ACRLKQAL   108
ScNsrR       EARRGRGGGLTLTDLGRRVSVGWLVRELEGEAEVVDCEG--DNPCPLRG---ACRLRRAL   111
GlBadM       KSFRGTGGGFLLAGPPESITLLQVVEAVEGPILPNRCVLKP-GECERDAS---CTVHPVW   112
TthNsrR      ESRVGRKGGVWPKLPPGEISLLKVMEALSGPVVLDLCATLKR--CPTEERRGFCYLKPGL   114
              :  *  **.        : :  ::.  .       *       *        * :

NgNsrR       GGAMKSFFTYLDGFTLQDLLNK--PTYDLLYESKIPIAVR-       147
RcNsrR       AGAQSQYYDFLNGYTLADCLRR--PRFLSPAP---------       140
BsNsrR       NEALMAYLAVLDNYTLRDLVKNKEDIMKLLRMKE-------       146
NeNsrR       SEALSAFMAVLDDYTLADLIAPRRQLSRKLHVMQISDSLSD       151
EcNsrR       SKAVQSFLTELDNYTLADLVEENQPLYKLLLVE--------       141
ScNsrR       RDAQEAFYAALDPLTVTDLVAAPTGPVLLGLTDR-PSG---       148
GlBadM       RQVQQQVRSILAGITLKDLATL-------------------       134
TthNsrR      ARTGLEIRKALAGLTLKDLLPENPPGA--------------       141
               .       *   *: *
```

Figure S1. Sequence alignment of NsrR[Th] with NsrR family members. The DNA-binding HTH domain is underlined, and conserved cysteines and glutamic acid responsible for the coordination of the iron-sulfur cluster in other NsrR members in gray. Note that the E residue conserved in all the NsrR homologs is absent in NsrR[Th]. Cysteine labeled in red was replaced in the C93A mutant. NgNsrR (YP_208569) from *Neisseria gonorrhoeae* FA 1090. RcNsrR (AAQ18178) from *Rhodobacter capsulatus*. BsNsrR (AEP85883) from *Bacillus subtilis* subsp. *spizizenii* TU-B-10. NeNsrR (NP_841002) from *Nitrosomonas europaea* ATCC19718. EcNsrR (NP_418599) from *Escherichia coli* K-12 substr. MG1655. ScNsrR (NP_632476.1) from *Streptomyces coelicolor* A3(2). GlBadM from *Geobacter lovleyi* SZ (YP_001951483). TthNsrR from *T. thermophilus* PRQ25 (FN666415).

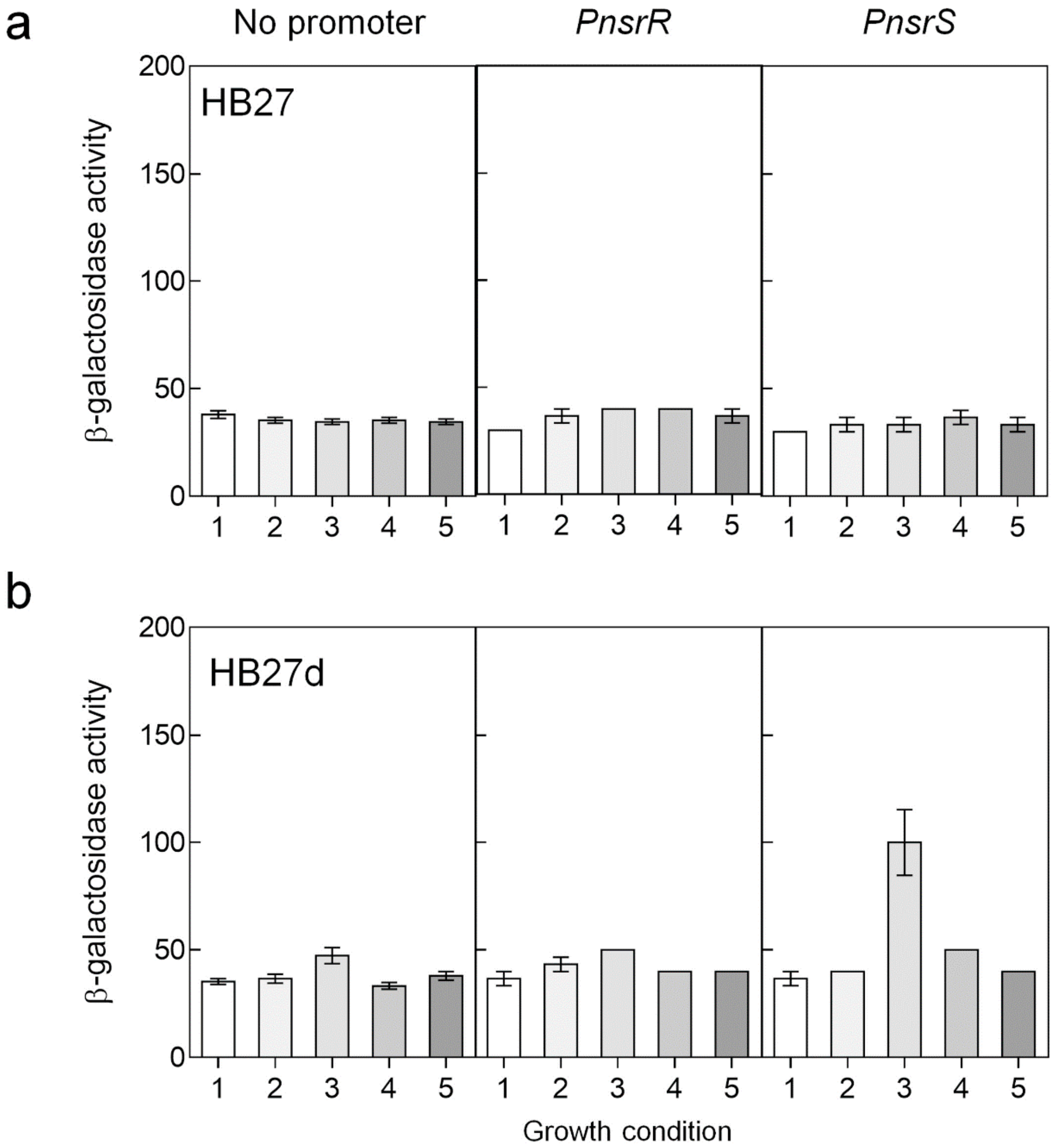

Figure S2. Transcriptional activity from the putative promoters of the *nsrR*, *nsrS* and *nsrT* genes. *β*-galactosidase activity was measured both in the obligate HB27 (**a**) and its denitrifying derivative HB27d (**b**) carrying the promoter probe plasmids pMHPnsrRbgaA (*PnsrR*, 303 bp), pMHPnsrSbgaA (*PnsrS*, 311 bp) or the empty plasmid pMHbgaA (no promoter). Transcriptional activity was measured in aerobic cultures (1) or after induction for 16 h under anaerobic conditions in the absence (2) or presence of 20 mM nitrate (3), 5 mM nitrite (4), or 100 M SNP (5). *β*-galactosidase activity is expressed as nanomoles of *o*-nitrophenol produced per min and per mg of protein. Data represent mean values from triplicate samples in at least two independent experiments; bars indicate standard error.

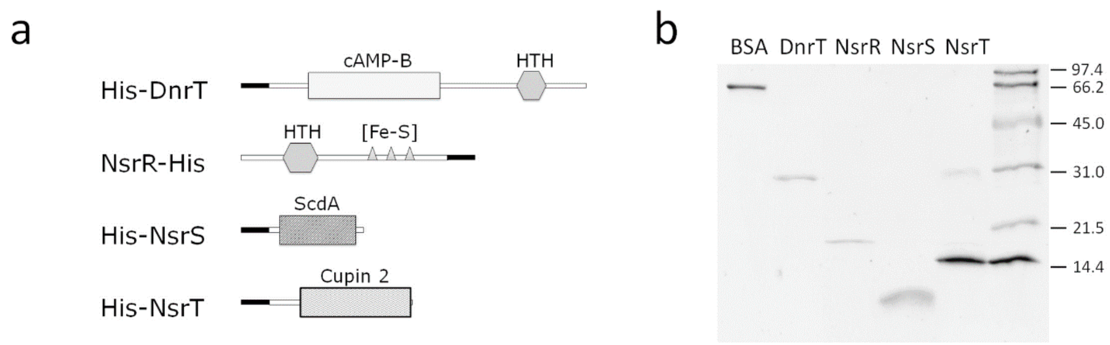

Figure S3. Production of recombinant His-tagged proteins. (**a**) Domains identified in DnrT, $NsrR^{Th}$, NsrS and NsrT. (**b**) SDS-PAGE of purified proteins after Ni-NTA affinity chromatography purification. The theoretical mass of each protein (in kDa) are: DnrT (27.0), $NsrR^{Th}$ (17.3), NsrS (9.1) and NsrT (12.6). 500 ng BSA was used as a loading control.

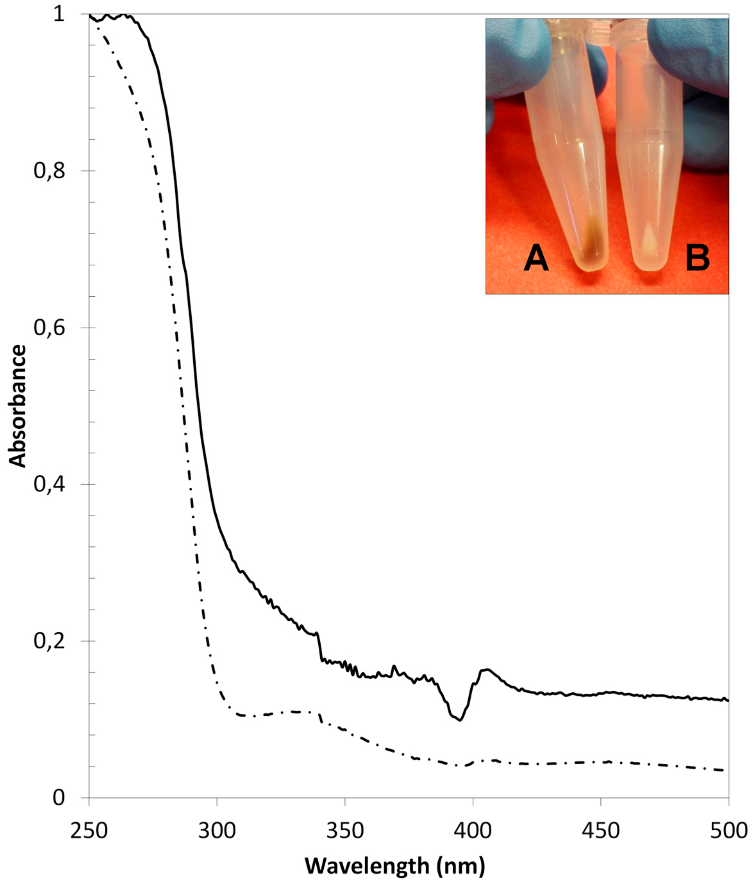

Figure S4. Production and spectroscopic analysis of $NsrR^{Th}$ and its C93A mutant. Recombinant His-tagged $NsrR^{Th}$ (A) and $NsrR^{C93A}$ (B) were overexpressed in *E. coli* BL21 and purified. Photographs of the culture pellets are shown in the right upper panel. The UV-visible spectrum of the corresponding purified proteins is shown. Dashed line corresponds to $NsrR^{C93A}$ and continuous line to $NsrR^{Th}$.

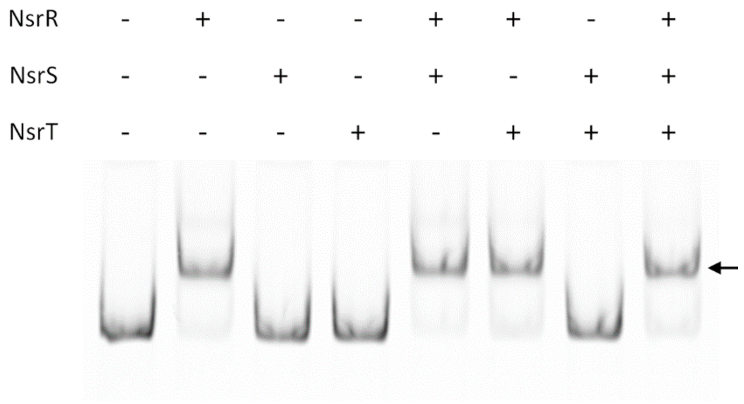

Figure S5. Effects of NsrT and NsrS on the binding of NsrRTh to the *PnorC* promoter. EMSA assays were performed with *PnorC* in the presence (+) or absence (-) of the indicated proteins at a protein:DNA molar ratio of 1:75. Arrows indicate the specific DNA-protein complex; bands at the bottom show unbound free promoter.

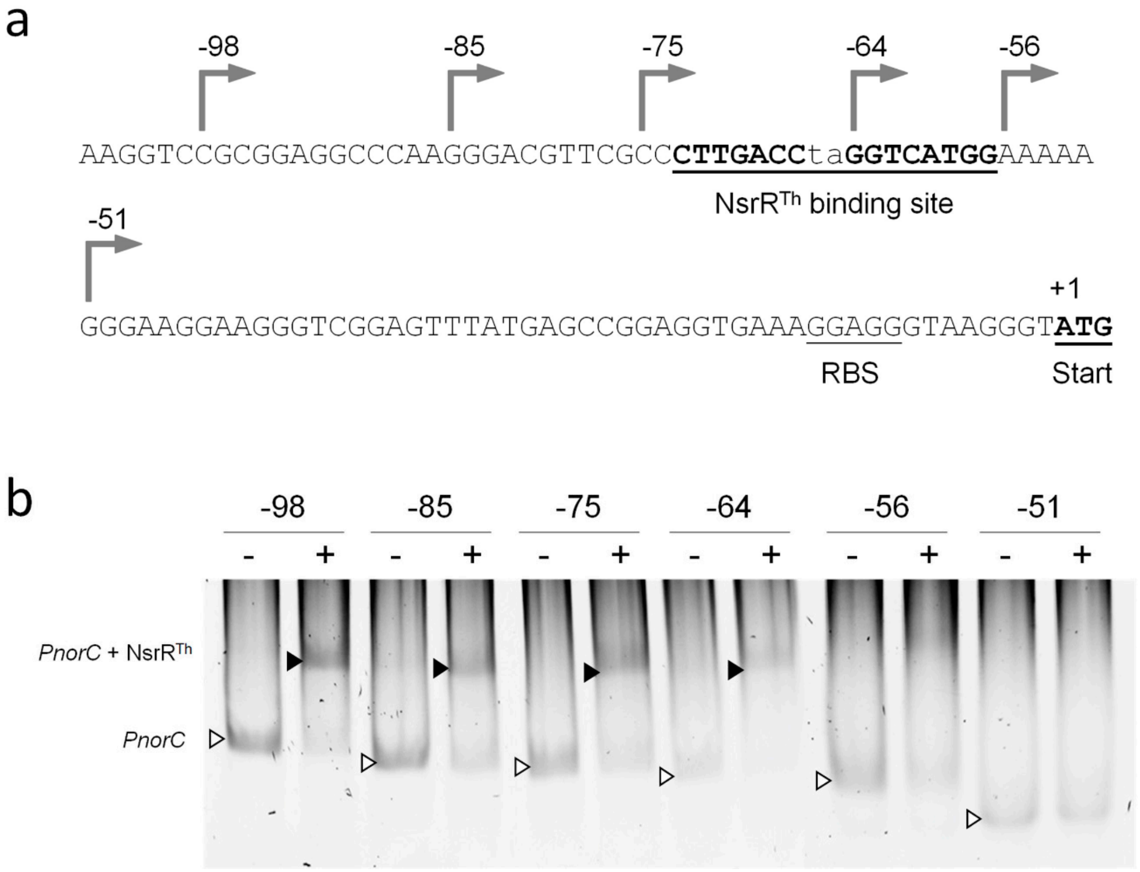

Figure S6. NsrRTh binds to a conserved palindromic sequence. (**a**) Scheme of the *PnorC* promoter and fragments used to test the NsrRTh binding capacity. Numbers indicate the 5′ end of the promoter fragments relative to the translation start codon. The putative NsrRTh binding site, the ribosome binding site (RBS) and the ATG codon are also indicated. (**b**) EMSA assays on *PnorC* fragments. Promoter probes (50 nM) were incubated in interaction buffer without (-) or with (+) NsrRTh at a 1:10 ratio for 10 min at 60 ºC. White and black arrows indicate the unbound or bound DNA respectively.
